# Good and Bad Sleep in Childhood: A Questionnaire Survey amongst School Children in Southern Italy

**DOI:** 10.1155/2011/825981

**Published:** 2011-12-21

**Authors:** Gianluca Ficca, Francesca Conte, Vittoria De Padova, Iole Zilli

**Affiliations:** ^1^Department of Psychology, University of Naples II, Via Vivaldi 43, 81100 Caserta, Italy; ^2^Department of Psychology, University of Florence, Via di San Salvi 12, Padiglione 26, 50135 Firenze, Italy

## Abstract

Despite its clinical importance, the issue of subjective sleep quality in children remains unexplored. Here we investigate, in school-aged children, the prevalence of bad sleep perception and its relationships with sleep habits and daytime functioning, to provide hints on its possible determinants. Subjective sleep perception, sleep habits, and daytime functioning were studied through a questionnaire survey in a sample of 482 children (6–12 yrs.). Being “bad sleeper” was reported by 6.9% of the sample. Compared to the “good sleepers”, these subjects displayed shorter sleep duration on schooldays, longer sleep latencies, and a more pronounced evening preference, beyond more frequent insufficient sleep. Though no differences emerged in sleepiness, bad sleepers showed higher impairments in daytime functioning, indicated by more frequent depressed mood and impulsivity. These distinctive features might be very important to precociously detect those children who are possibly more vulnerable to sleep disturbances and whose sleep-wake rhythms evolution should be paid particular attention thereafter.

*“The good people sleep much better at night than the bad people.*

*Of course, the bad people enjoy the waking hours much more”*

Woody Allen

## 1. Introduction

Good sleep is extremely important for all aspects of health and wellbeing in humans.

But what exactly does “good sleep” mean? A first basic distinction should be made between those characteristics that can be considered “objective” indicators of sleep quality and the subjective perception of sleeping well.

The latter is extremely important in itself since subjective sleep complaints, which are the most frequent reason for seeking medical help, are not necessarily concordant with what objectively measured, as highlighted by a number of studies, conducted both in normal [1, 2] and pathological populations [3–5], and in different age groups [6, 7].

In children, the first question that still requires a definite answer is how many of them believe their sleep is good. In fact, data on the prevalence of good sleep perception are scarce over the whole life span.

Most of the available data concerns the adult population: in 2005, the USA National Sleep Foundation [8] reported that 26% of the adult population claim that they have “a good night's sleep” only a few nights a month or less. Previous European surveys have reported the presence of sleep dissatisfaction in 10.1% of the Italian population [9], 11.9% of the Finnish [10], 10.1% of the Portuguese [11], and 7% of the German [12].

In their classical paper on subjective sleep quality in aging, Buysse and colleagues [13] found that almost 70% of people aged more than 80 fell within a categorically defined range for “good” sleepers.

As for younger populations, data on subjective sleep quality perception are even scarcer. Despite a number of surveys in children and adolescents reporting a high prevalence of sleep problems, such as night awakenings [14], nightmares [14], nocturnal enuresis [15], and sleep-onset delay [16], the question of the global subjective perception of sleep quality in childhood has been almost totally neglected. Indeed, only one study on 449 toddlers has directly assessed sleep satisfaction in this population, by means of a question on their global sleep quality perception [17]. Mediocre or bad quality of sleep was reported by 22.4% of the sample. However, in this research, subjective perception was only taken into account as one of the factors, the others being objective sleep features, contributing to a global “quality of sleep” score.

Both in the clinical and the experimental fields [18], optimal sleep duration, sleep continuity, and sleep organization are considered main indicators of objective sleep quality [19]. These features do not necessarily correspond to those required for subjective judgments of good sleep.

Actually, subjective determinants of sleep quality perception have been only occasionally studied in the adult individual. Among sleep features it seems that sleep continuity [20] and the ease of sleep onset [21] play a pivotal role, but perceived depth of sleep [22] and sleep duration [23] have been proposed as relevant determinants as well. Concerning waking features, ease of waking [24], freshness on waking [25, 26] and throughout the day [24, 25] are the factors appearing to give the greater contribution.

However, there is very little systematic knowledge on factors underlying sleep satisfaction judgments both in children and in the elderly and on whether these determinants might exhibit age-related differences. Indeed, only one recent work by Zilli and coworkers [7] was carried on elderly subjects—who were shown to evaluate their own sleep quality as mainly dependent on sleep latency and length rather than on continuity and thus to maintain a perception of good sleep despite the high number of awakenings—and not even one study on this topic has so far regarded childhood.

Finally, daytime functioning in children has been investigated in relation to objective measures of sleep or self-reported sleep habits and problems: in these studies, a number of sleep features such as degree of fragmentation [27], sleep duration [28], difficulties of falling asleep [29], and sleepiness [30] have been shown to relate to children daytime functioning and school performance. However, again, due to the lack of data on subjective sleep quality, it remains to be ascertained whether and to what extent sleep satisfaction in children could be related to the quality of their waking.

Thus, aims of this study are:

(1) to determine the prevalence of sleep satisfaction in a sample of children in Southern Italy;

(2) to investigate how dissatisfied children differentiate from satisfied ones as far as sleep habits, subjective evaluation of sleep characteristics, circadian preference, vigilance levels, and daytime functioning are concerned.

## 2. Materials and Methods

### 2.1. Subjects

The survey has been carried out from May to June 2009 in three public Elementary Schools, respectively located in S. Nicola La Strada (Caserta), Naples, and Pomigliano d'Arco (Naples). These schools were randomly selected from the list of all Elementary Schools in Campania, a wide region of southern Italy.

Headmasters of the selected schools were first contacted through a formal letter, introducing the research and the professionals involved. For those who positively answered, all the procedures, instruments, and aims of the study were explained in a further meeting, extended to teachers and parents' representatives. A final meeting served to illustrate the study to all children's parents and to collect their informed consent.

Four hundred eighty-two students were recruited for the study, the only exclusion criterion being the presence of a diagnosed cognitive or learning disorder.

Table 1 displays the demographic characteristics of the sample.

### 2.2. Instrument

The instrument used in the study is the School Sleep Habits Survey [31–34] in its Italian version [35–37]. The questionnaire includes the following:

1. questions assessing sleep habits on both schooldays and weekends: three of these are open questions, addressing sleep duration and bedtime and rise time in hours and minutes; two others, investigating the reasons for going to bed and rising at a given time, are multiple choice questions with seven predetermined answers;
2. one question on habitual sleep latency, consisting of a single forced-choice item with six response categories (“0 to 5 minutes”, “6 to 15 minutes”, “16 to 30 minutes”, “31 to 45 minutes”, “46 to 60 minutes”, “more than one hour”);
3. a context and vigilance scale, composed of nine items assessing the ease of staying awake in different situations (“talking *vis a vis *with someone else”, “travelling on public transports”, “watching a show”, “watching television or listening to music”, “reading or studying”, “during a school test”, “sitting in class”, “working at the computer”, “playing a videogame”). Respondents had to choose among four ordinal alternatives, ranging from “no difficulty staying awake” to “struggling to stay awake but falling asleep”. A global vigilance score was then obtained by summing up scores at all of the nine items;
4. a sleep-wake behavioural problems scale, made up of 17 items which assesses, over the last two weeks, how often the subjects have experienced some sleep*∖*wake-related perceptions and problems, (“being happy about one's sleep”, “being late at school for having slept too long”, “falling asleep in class”, “waking up too early without being able to go back to sleep”, “going to bed late in the evening”, “staying awake all night long”, “sleeping until noon”, “feeling tired or sleepy during the day”, “having difficulties waking up in the morning”, “having problems falling asleep at bedtime”, “having nightmares”, “going to bed too early because of excessive sleepiness”, “doing something dangerous”, “sleeping well”, “feeling sad or depressed”, “feeling anxious or nervous”, “feeling very worried”). The answers are graded on a five-point scale, ranging from “never” to “always”;
5. a morningness-eveningness questionnaire (MEQ), composed of ten items assessing circadian preference to discriminate subjects in morning types (M-types), intermediate types (I-types), and evening types (E-types).
6. Finally, an item about sleep quality perception (“Do you consider yourself as a good or a bad sleeper?”) was added to the original Italian version of the questionnaire [35], as well as a forced question examining whether sleep duration is considered sufficient: (“How often do you think you sleep enough?”, with five choices: “always”, “often”, “sometimes”, “seldom”, “never”).

### 2.3. Procedure

Questionnaires were administered by an experimenter, in presence of the teachers, during school hours. In view of the subjects' young age, the experimenter remained in the classroom throughout the administration procedure, being available to answer any question arising during the questionnaire completion. He was specifically instructed to provide standardized answers, which included question rephrasing and examples.

### 2.4. Data Analysis

After descriptive statistics, the global sample was split in two groups (“good sleepers” and “bad sleepers”), based on the answer to the question assessing overall subjective sleep quality. The prevalence of good and bad sleepers was calculated for the total sample, for males and females separately, and, to detect age-related differences, within two different age groups: 6–8 and 9–12 years.

Good and bad sleepers were then compared for the following dependent variables:

- sleep habits, that is, sleep duration in minutes, bedtime and rise time—all variables reported for schooldays and weekends—plus the differences between schooldays and weekends (Δ-SD/WE) in bedtime, rise time, and sleep duration;
- sleep latency;
- sufficiency of sleep;
- circadian preference, calculated by summing up scores at the ten questions (lower scores being associated to a higher degree of eveningness);
- vigilance, calculated by summing up scores at the nine questions ranging from 1 to 4, so to have a maximum score of 36 and a minimum score of 9;
- sleep-wake behavioural problems, calculated by summing up scores at the seventeen questions ranging from 1 to 5 (higher scores corresponding to lower degrees of problems).

For comparisons, nonparametric Mann-Whitney *U*-test was used for all cardinal variables.

Chi-square test was carried for all binomial variables, as well as for analysis of frequency distribution of chronotypologies.

Furthermore, Pearson's analysis of correlation was carried between the overall sleep-wake behavioural problems score and sleep habits measures.

## 3. Results

### 3.1. Response Rate

The questionnaire was correctly filled by all children (*N* = 482). However, seventeen questionnaires had to be excluded for aberrant values. Thus, the final sample for data analysis included 465 children (*F* = 231, *M* = 232, two children did not report their sex; age range 6–12 years, 6–8 years: *N* = 149, 9–12 years: *N* = 316). 

### 3.2. Overall Sleep Quality

**Figure 1:**
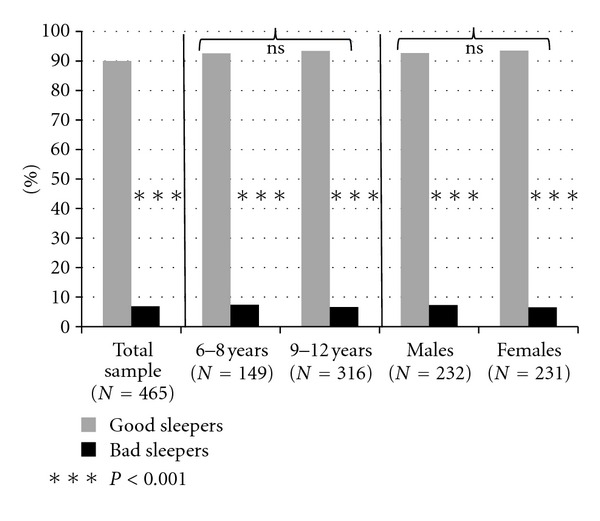
Prevalence of good and bad sleepers in the total sample, in two age groups (6–8 and 9–12 years), and across genders.

To the question about global sleep quality, 6.9% of the sample answered to be “bad sleepers”, whereas 90% answered to be “good sleepers”, and 3.1% did not answer (Figure 1). Bad sleepers percentages did not significantly change either across age (6–8 years: 7.4%, 9–12 years: 6.6%, chi^2^= 0.07, ns) or across genders (*M*: 7.3%, *F*: 6.5%, chi^2^ = 0.08, ns).

### 3.3. Sleep Habits and Sleep Features

Table 2 displays sleep onset time, rise time, and sleep duration in the general sample, as well as the results of the comparison for the same variables between good and bad sleepers. Sleep onset time is significantly delayed for bad sleepers both during schooldays (*P* = 0.025) and weekends (*P* = 0.027). Furthermore, sleep duration is significantly shorter in bad sleepers compared to good sleepers in schooldays (*P* = 0.028). No significant differences were found in either rise time or sleep duration between bad and good sleepers during schooldays and weekends.

Also, the two groups did not significantly differ in the Δ-SD/WE of bedtime (*U* = 6937.0, ns), rise time (*U* = 5688.5, ns), and sleep duration (*U* = 5762.5, ns).

### 3.4. Sleep Latency and Sufficiency of Sleep

Sleep latency is significantly longer in bad sleepers (good sleepers median = 2, that is, “6 to 15 minutes”; bad sleepers median = 4, that is, “31 to 45 minutes”; *U* = 4425.0, *P* < 0.001). Moreover, bad sleepers report more frequent insufficient sleep (good sleepers median = 4, that is, “seldom”, bad sleepers median = 3, that is, “sometimes”; *U* = 5403.0, *P* = 0.034).

### 3.5. Circadian Preference

**Figure 2:**
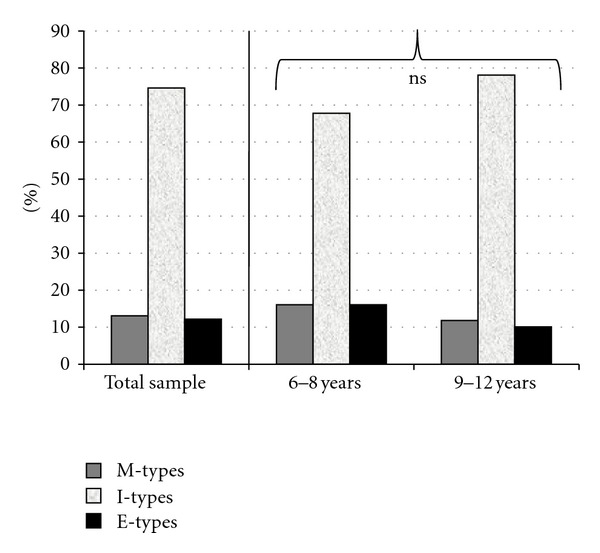
Frequency distribution of chronotypologies in the total sample and in two age groups (6–8 and 9–12 years).

As shown in Figure 2, frequencies of M-types, I-types, and E-types were, respectively, 10.1%, 74.6%, and 12.2%. No significant differences emerged in frequency distribution between the two age groups considered (chi^2^ = 4.53, ns).

Significant differences were found in MEQ global scores between good and bad sleepers (*U* = 4088.5, *P* = 0.003), with bad sleepers more frequently displaying an evening preference.

### 3.6. Context and Vigilance

No significant differences between bad and good sleepers were found either in the context and vigilance global score (*U* = 5844.5, ns) or in any of the items composing the scale.

### 3.7. Sleep-Wake Behavioural Problems

A significant difference emerged between bad and good sleepers at the sleep-wake behavioural problems global score (*U* = 3575.5, *P* < 0.001), displayed in Table 3 together with the comparisons for all of the specific items showing a significant difference.

In addition, the global score in the overall sample showed significant correlations with rise time both in schooldays (*r* = −.095, *P* < 0.001) and weekends (*r* = −.122, *P* = 0.02), whereas it was not significantly correlated with bedtime (schooldays: *r* = −.095, ns; weekends: *r* = −.087, ns), sleep duration (schooldays: *r* = −.003, ns; weekends: *r* = −.003), or Δ-SD/WE measures (bedtime: *r* = −.042, ns; rise time: *r* = −.056, ns; sleep duration: *r* = −.002, ns).

## 4. Discussion

At best of our knowledge, this is the first study addressing subjective sleep quality perception in school-aged children and may contribute to trace the entire life-span trajectory of subjective sleep quality ratings and of their determinants.

In order to address *habitual* sleep in a relatively large sample, we decided to collect only subjective data by means of a standardized questionnaire with good reliability and validity properties [31–34], already confirmed in the Italian population [35–37]. However, at a further stage, these data might be complemented by objective sleep measures in smaller samples, over repeated consecutive nights.

As for our choice of directly asking for the perception of both sleep quality and sleep features to the children themselves, it was made at variance with the vast majority of the studies on children younger than 10 years, which have been based on parents' and teachers' reports. We are confident that this choice may be well rewarding. In terms of feasibility, our questionnaire was filled out by all children, without any specific difficulty in data collection. As for accuracy, we have taken into account the severe limitation of parent-reports studies represented by the scarce concordance between adults' perceptions and children's objective sleep parameters [38, 39]; in fact, parents appear to be accurate in reporting sleep schedule measures (sleep onset time, time in bed) but they tend to overestimate the duration and continuity of their children's sleep and therefore its global quality [39–41]. Also, parent-child agreement on the report of night-time awakenings was recently found to be remarkably low, and even lower was the concordance on overall subjective sleep quality [42]. Finally, Owens and coworkers [43] found that the differences in actigraphic sleep parameters between ADHD and control children were more often correlated with self-reports than with parent reports, thus emphasizing the importance of directly questioning the school-aged child.

In addition, though the reliability of self-reports in children at very early ages (6–8 years) could be questioned, this is not the first study concerning sleep habits that used self-reports in this age group [40, 44, 45].

A first major result of the present survey is the rather low percentage (6.9%) of school children claiming to be bad sleepers. We did not find any comparable data in the literature, since in the few studies on the same age group administering a question on subjective sleep quality, this question was part of a sleep log, thus referring to a specific night instead of assessing the subjective perception of habitual sleep [42–44].

Regarding sex, no difference in the percentage of bad sleepers was detected between males and females, at variance with data on adolescence, showing that subjectively assessed sleep quality indicators are better in girls than boys [46], and with studies on adult populations, reporting, conversely, higher percentages of sleep complaints in women [47], despite better objective sleep measures [48].

As for age differences, the prevalence of subjectively reported bad sleep in our group of children is lower than the one previously found both in adults [8–11] and old subjects [13], but almost identical to data from an Iranian study on high school students [49]. Together with the evidence that we did not find any significant difference of bad sleepers percentage when comparing two age subgroups within the whole sample, this might suggest that the prevalence of bad subjective sleep quality increases only after adolescence. A possible reason for this will be made clearer later on, after discussing our results on sleepiness levels and daytime behavioural problems.

In our sample, bad sleepers' sleep duration was lower than that of good sleepers on schooldays. Also, they reported insufficient sleep more often than good sleepers and longer sleep latencies, as well as delayed sleep onset time on both schooldays and weekends.

Furthermore, the bad sleepers group displayed a higher global score on the sleep-wake behavioural problems scale. In particular, it is noteworthy that bad sleepers reported significantly higher scores at the items “have done something dangerous” and “have felt sad and depressed”, suggesting a somehow more impulsive attitude and a tendency to depressed mood.

Both the previously mentioned data about sleep features and the presence of a poorer daytime functioning compared to good sleepers might be explained by looking at chronotypologies through the morningness-eveningness questionnaire results. In general terms, we have detected an equal frequency of evening and morning types in the total sample—slightly above 10%, similar to the one of the adult population—and no differences between our two age groups, which range from elementary schoolchildren (6–8 years) to prepubertal children (9–12 years). This suggests that the high frequency of eveningness, often observed in adolescence [35, 46], is peculiar of that specific age and not preceded by a gradual shift towards it.

However, what is most interesting for our purposes is our data concerning a clear evening preference for bad sleepers. This result is consistent with Giannotti and coworkers' finding [37] of a high prevalence of self-reported poor sleep in evening-type (E-type) adolescents (about one third of the E-type group), and with the higher presence of sleep-wake behavioural problems in E-types relative to other chronotypes [35, 37]. Indeed, as also noted by Russo and colleagues [35], the higher prevalence of sleep-related problems in E-types could be explained as a result of “social jet-lag”, a concept introduced by Wittmann and colleagues [50] to indicate the misalignment between individual biological rhythms and the social rhythm imposed by school schedule.

On the other hand, no difference emerged between bad and good sleepers' scores on the context and vigilance scale. Though in contrast with the negative influence of sleep disturbances on daytime sleepiness, described in most of the literature (see e.g., [51, 52]), the possibility that the reported sleep problems do not impact daytime vigilance levels receives support from a previous study on an adult population [53], in which E-types, who presented repeated episodes of sleep restriction and irregular sleep-wake schedules, did not rate themselves as sleepier than morning and normal chronotypes.

Moreover, in a very recent meta-analysis on children's sleep and cognition, after publication bias correction—a necessary step in the meta-analytic process—total sleep time and sleep efficiency displayed no correlation with daytime sleepiness, whereas the relationship between sleep and behaviour was indeed significant (Rebecca Astill, personal communication).

In light of these results, we can trace two hypotheses on the determinants of poor sleep perception in our bad sleepers group. On one hand, it is possible to consider their sleep quality judgments as the result of objectively worse sleep features, which would on their turn influence mood and impulsive attitudes though not impacting perceived alertness. If this is the case, sleep duration and latency are possibly the main determinants, as confirmed by the finding that bad sleepers report perception of insufficient sleep and difficulties falling asleep more frequently than good sleepers, similarly to what observed in the elderly [7].

Yet, these differences might be negligible although significant, and bad sleep perception could be the consequence of additional factors other than sleep itself. In the first place, as proposed by some authors [25, 54], sleep satisfaction judgments could be partly or fully determined by cognitive biases. In other words, the presence during waketime of specific emotional and behavioural problems, such as depressed mood and impulsivity, may lead to retrospectively define sleep quality as poor and unsatisfactory. Moreover, as mentioned above, an important role may be played by “social jet lag”, although no difference in “social jet-lag measures” between good and bad sleepers was found in our sample.

This alternative view might also provide an answer to the aforementioned question on the increase in the prevalence of poor sleep perception occurring from adolescence onward. The main factor interfering with judgments on sleep quality would actually be perceived wake quality, which is often dramatically worsened at that age as a result of many factors, including modified life styles and more challenging social and work demands.

On the basis of our evidence, it would be hazardous to privilege or discard any of these two major hypotheses, which are likely to be not mutually exclusive and to interact in determining children's poor sleep judgments, as it was shown to happen in insomniac adults as well [25, 54].

In conclusion, the subjective perception of sleep characteristics and daytime behavioural features seems quite peculiar in the group of children reporting themselves as bad sleepers. This observation is of great clinical interest, in that it strongly suggests a possibility to set specific strategies aimed to screen and precociously detect those subjects who might be more vulnerable than others to sleep disturbances over the life span.

## Figures and Tables

**Table 1:** Demographic characteristics of the sample.

	*N *	*M *	*F *	6–8 years	9–12 years
Total sample	482	240*	240*	164	318
S. Nicola La Strada	117	52	65	26	91
Naples	83	40	43	31	52
Pomigliano d'Arco	282	148	132	107	175

*Summing up the number of subjects in the males and females groups does not yield the total sample number since two children did not report their sex on the questionnaire.

**Table 2:** Descriptive data on sleep habits and their comparison between good and bad sleepers.

Variables	Total sample Mean ± SD	Good sleepers Mean ± SD	Bad sleepers Mean ± SD	Mann-Whitney test (*U*)
Sleep onset time on schooldays	21:46 ± 01:23	21:43 ± 01:22	22:22 ± 01:24	5308,0*
Sleep onset time on weekends	22:53 ± 02:08	22:50 ± 01:30	23:35 ± 02:10	5322,0*
Rise time on schooldays	07:20 ± 00:31	07:21 ± 00:32	07:18 ± 00:24	6652,0
Rise time on weekends	09:25 ± 01:31	09:23 ± 01:28	09:47 ± 02:01	5838,50
Sleep duration on schooldays	09:34 ± 01:24	09:37 ± 01:23	08:55 ± 01:25	5330,0*
Sleep duration on weekends	10:32 ± 02:30	10:33 ± 02:29	10:12 ± 02:37	6715,5

*P < .05.

**Table 3:** Comparison of sleep-wake behavioural problems between good and bad sleepers.

	Good sleepers			Bad sleepers			
Variables	Median	1° Quartil	3° Quartil	Median	1° Quartil	3° Quartil	*U* of Mann-Whitney Test
Have been happy about your sleep	4	4	4	3	1,5	3	4339,5***
Have had problems falling asleep	4	3	4	2	1,75	2	4073***
Have had a nightmare	4	2	4	2	2	2	5158**
Have done something dangerous	5	3	5	4	2	4	5216,5**
Have slept well	5	4	5	4	1,75	4	3242,5***
Have felt sad or depressed	5	3	5	3	2	3	5012,5**
Sleep-wake behavioural problems global score	67	60	67	58	50,5	58	3575,5***

*P < .05.

**P < .01.

***P < .001.
